# Bibliometric Analysis of Biopolymer-Based Materials in Wastewater Treatment

**DOI:** 10.3390/polym18080953

**Published:** 2026-04-14

**Authors:** Anathi Dambuza, Pennie P. Mokolokolo, Mamookho E. Makhatha, Motshabi A. Sibeko

**Affiliations:** 1 Department of Chemistry, University of the Free State (QwaQwa Campus), Kestell Road, QwaQwa, Phuthaditjhaba 9866, South Africa; anathidambuza@yahoo.com; 2 Department of Chemistry, Rhodes University, Prince Alfred Road, Makhanda 6139, South Africa; petrus.mokolokolo@ru.ac.za; 3 Department of Metallurgy, University of Johannesburg, Doornfontein Campus, 55 Beit St, Doornfontein, Johannesburg 2028, South Africa; emakhatha@uj.ac.za

**Keywords:** bibliometric analysis, chitosan, wastewater treatment, biopolymer, pollutants

## Abstract

Biopolymer-based materials have gained significant attention as sustainable alternatives for wastewater treatment due to their biodegradability, low toxicity, and high adsorption potential. Despite extensive research on individual materials such as chitosan, cellulose, and alginate, a comprehensive synthesis of global research trends integrating multiple biopolymers remains limited. This study addresses this gap through a large-scale bibliometric analysis of 13,720 publications indexed in the Scopus database from 1995 to 2025. The dataset was systematically analysed using VOSviewer to evaluate publication trends, leading journals, countries, institutions, collaboration networks, and keyword co-occurrence patterns. The results reveal a rapid growth phase after 2016, driven by increasing global demand for sustainable water treatment technologies. China, India, and the United States emerged as the most productive and influential contributors. Keyword analysis highlights adsorption, wastewater treatment, cellulose, and chitosan as dominant research themes, with growing emphasis on composite materials and functional modifications. Beyond descriptive metrics, this study identifies key research gaps, including limited focus on scalability, regeneration efficiency, and real-world application of biopolymer-based systems. The findings provide a comprehensive understanding of the evolution and current direction of the field, offering strategic insights for future research and development of high-performance, sustainable wastewater treatment materials.

## 1. Introduction

The growing environmental and public health concerns caused by water pollution throughout the world have made research on biopolymer-based materials (e.g., chitosan, alginate, cellulose, agar, and starch) in wastewater treatment an essential field of study [1,2]. Over recent decades, the field has progressed from fundamental adsorption studies to sophisticated composite materials and nanotechnologies, reflecting a trajectory marked by growing research output and diversification of applications [3,4]. These biopolymers are intriguing options for sustainable water remediation because of their biodegradability, renewability, and functional versatility [5,6]. The global need to address contaminants such as heavy metals, dyes, pharmaceuticals, and emerging organic pollutants highlights the practical relevance of this study, with millions of litres of wastewater requiring effective treatment each year [7,8]. Despite significant research, obstacles remain in optimising the practical application of these biopolymers for wastewater treatment [9,10]. The problem centres on limitations such as mechanical stability, regeneration capacity, and scalability of biopolymer-based materials [11,12]. Although researchers have documented the adsorption capabilities and modification techniques of biopolymer-based materials, thorough bibliometric evaluations that integrate trends, gaps, and collaborative networks across materials remain fragmented [4,6,13]. This is because these studies are generally material-specific or application-specific, limiting their ability to provide a holistic perspective on the broader field of biopolymer-based wastewater treatment. As a result, comparative insights across different biopolymers, their functional modifications, application domains, and global research networks remain insufficiently explored. The balance between material performance and environmental effect remains a source of dispute, with some research emphasising laboratory-scale efficacy and others highlighting real-world implementation challenges [14,15]. The lack of a comprehensive synthesis of global research hinders the identification of key knowledge gaps and the translation of laboratory discoveries into scalable technologies [16,17].

Therefore, this study aims to conduct a comprehensive cross-biopolymer bibliometric analysis of global research on biopolymer-based materials for wastewater treatment. By analysing a large dataset of publications indexed in the Scopus database (1995–2025) using bibliometric tools such as VOSviewer [18,19], this work seeks to identify publication trends, leading contributors, collaboration patterns, and key research themes. Beyond descriptive metrics, the study provides integrated insights into research development, knowledge gaps, and future directions, thereby contributing to a deeper understanding of how biopolymer-based technologies can be advanced toward sustainable and scalable wastewater treatment solutions.

## 2. Types and Some Sources of Biopolymers

**Figure 1:**
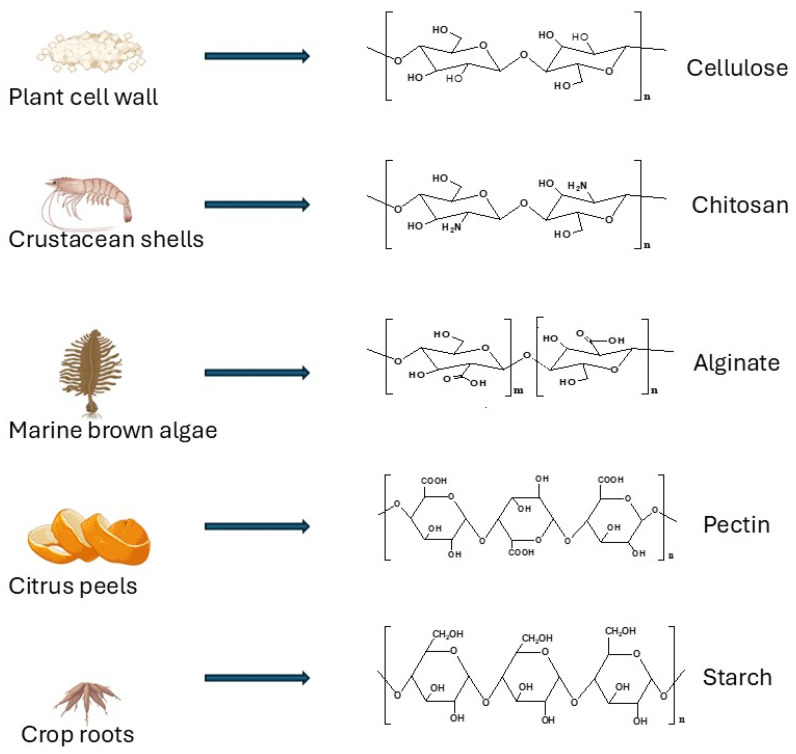
Different biopolymers that are used in wastewater treatment and their sources.

This section provides a concise contextual overview of the major biopolymers identified in the bibliometric analysis. It is not intended as a systematic but rather as an illustrative summary to support the interpretation of bibliometric trends observed in the dataset. Biopolymers have emerged as a viable solution for wastewater treatment, providing an environmentally friendly alternative to traditional chemical treatments. These materials, derived from natural sources (see Figure 1), are biodegradable, non-toxic, and cost-effective, making them ideal for tackling the environmental concerns faced by industrial and municipal wastewater [20]. Biopolymers are used in wastewater treatment to remove a variety of contaminants, including heavy metals, dyes, and emerging organic pollutants, through methods such as adsorption, flocculation, and coagulation. These biopolymers include chitosan, alginate, cellulose, agar, and starch.

### 2.1. Cellulose

Cellulose is a linear polymer composed of β-D-glucose units linked by β-1,4-glycosidic bonds. It is the most prevalent biopolymer on Earth, predominantly found in plant cell walls. It is a structural polysaccharide that gives stiffness and strength to plant cells [21]. It can be obtained from a variety of sources, including plants, wood cell walls, algal tissues, and some types of bacteria [22]. Cellulose has unique properties, such as biodegradability, environmental friendliness, and biocompatibility, which make it an appealing material for various industrial uses, including wastewater treatment [23,24]. However, despite this biopolymer having these properties, its use in wastewater treatment is limited due to its chemical structure and physicochemical properties. The structure of natural cellulose has various hydrogen bonds inside and between molecules and is highly crystalline, which makes it insoluble in water and common organic solvents [25]. Therefore, its structure needs to be modified to meet its applications in wastewater treatment. Table 1 shows biopolymer-based materials, including cellulose, in wastewater treatment. It also demonstrates that the adsorption capacity of most biopolymer-based materials is governed by chemical functionalization and composite design, while native or minimally modified materials have low adsorption capacities.

It should be noted that the adsorption capacities reported in Table 1 are influenced by experimental conditions such as pH, initial concentration, contact time, and temperature. The table is intended to illustrate general trends rather than provide definitive performance rankings.

### 2.2. Chitosan

Chitosan is a linear polysaccharide that consists of β-(1→4)-linked D-glucosamine and N-acetyl-D-glucosamine units [52]. It is one of the most often utilised biopolymers in wastewater treatment, produced by the deacetylation of chitin, a polysaccharide present in crustacean shells (e.g., prawn, crab, and lobster exoskeletons), insects and fungi [53]. It has abundant amino (-NH_2_) and hydroxyl (-OH) groups, which help facilitate the adsorption of different pollutants through various mechanisms such as chelation, hydrogen bonding, and electrostatic attraction, enabling efficient removal of both cationic and anionic pollutants [54]. However, raw chitosan has shortfalls in that also has limited application in wastewater treatment due to attributes such as its poor solubility in neutral and basic pH and poor mechanical strength. So, it is often modified to enhance its properties [55,56].

### 2.3. Alginate

Alginate, also known as alginic acid, is a naturally occurring polysaccharide derived from marine brown algae. It is produced mostly from various Laminaria species, such as Laminaria Hyperborea, Laminaria Digitata, Laminaria japonica, and Macrocystis pyrifera. Some bacteria, including Azotobacter vinelandii and Pseudomonas aeruginosa, also generate it [57,58,59]. Alginate extraction relies on the water solubility of its sodium salt, as alginic acid is insoluble; therefore, calcium and potassium alginates are converted to sodium alginate using sodium carbonate. Natural alginate in various salt forms (e.g., calcium, magnesium, or other algal alginates) is converted into water-soluble sodium alginate during alginate extraction with aqueous alkali solutions (NaOH) [60]. Grafting, crosslinking, and blending can all be used to improve the characteristics of alginate for specific purposes. Such modifications can increase its metal binding and adsorption characteristics, making it useful for wastewater treatment [61].

### 2.4. Pectin

Pectin is a natural anionic polysaccharide mainly derived from the cell walls of fruits and vegetables, especially citrus peels and apple pomace, which are two important agro-industrial byproducts. Its structure mainly consists of α-(1→4)-linked D-galacturonic acid units. Some of these units are methyl-esterified at the carboxyl (-COOH) groups, resulting in various degrees of esterification (DE). The DE determines whether pectin is high-methoxyl (HM) or low-methoxyl (LM), affecting its gelation and metal-binding properties [62]. It can be chemically functionalized to improve its adsorption capacity, allowing for efficient removal of heavy metals, dyes, and other pollutants from wastewater. At the same time, its derivatives serve as flocculants, adsorbents, and components in hybrid materials and metal–organic frameworks for water purification, thereby promoting environmental sustainability and supporting circular economy practices by converting waste into eco-friendly treatment resources [63].

### 2.5. Other Biopolymers

Starch is a naturally occurring biocompatible and biodegradable biopolymer found in the stalks, roots, and crop seeds of several plants. Starch granules with 3D architecture have a crystallinity of 15% to 45% and include d-glucose units with bio-macromolecules such as amylopectin, branched (1→6) α-d-glucan, amylose, and linear (1→4)-linked α-d-glucan [64]. It has a unique structure that enables it to interact with a variety of contaminants via hydrogen bonding and hydrophobic interactions. This makes it an efficient adsorbent for heavy metals and emerging contaminants in wastewater treatment [12,65]. Other natural fibres, including lignin and agar, have been demonstrated to be a key remedy in wastewater treatment [66,67].

## 3. Materials and Methods

### 3.1. Document Collection

The document collection process for this study was performed using the Scopus database, one of the most comprehensive and globally recognised academic indexing platforms [68]. Using the advanced search query (chitosan OR alginate OR cellulose OR starch OR lignin OR pectin OR agar OR biopolymer AND in AND wastewater AND treatment) AND PUBYEAR > 1994 AND PUBYEAR < 2026, an initial 14,788 documents were retrieved. The search was performed using the advanced search option, targeting the title, abstract, and keywords fields to ensure relevance and consistency. To ensure consistency in the temporal evolution of biopolymer-based materials for wastewater treatment, the document set was filtered by publication year (1995–2025), yielding a refined dataset that reflects three decades of research progress. Only English documents were included in this study, and the document types included research articles, review articles, conference papers, conference reviews, book chapters, and short surveys.

After applying these inclusion criteria, the final number of documents was 13,720, representing the core dataset used for bibliometric and scientometric analysis. These documents were exported in CSV format, which is compatible with VOSviewer, enabling the construction of collaboration networks, keyword co-occurrence maps, and citation impact visualisations. The analysis revealed that the dominant subject area, by having many documents, was environmental science (6844), highlighting the strong environmental focus of biopolymer applications in wastewater treatment. This was followed by chemical engineering (4124), chemistry (3962), materials science (3081), engineering (2820), and biochemistry, genetics, and molecular biology (2277), demonstrating the multidisciplinary nature of research in this field.

The curated dataset was then utilized to examine annual publication trends, citation performance, subject area distribution, and international collaborations, providing a complete picture of scientific activity and new directions in biopolymer-based wastewater treatment research.

### 3.2. VOS Viewer Analysis

VOSviewer version 1.6.20 was used to analyse the lists of authors, institutes, and countries. Van Eck and Waltman (2010) developed a program for creating and displaying bibliometric networks [69]. For network construction, the full counting method was employed, where each co-authorship or keyword occurrence was assigned equal weight. Data normalisation was performed using the association strength method, which is the default normalisation technique in VOSviewer and allows for accurate mapping of relationships between items.

Threshold values were applied to ensure meaningful visualisation and to reduce noise in the network maps. For example,

- A minimum number of documents per author was set (e.g., ≥20 publications) for co-authorship analysis.
- A minimum number of occurrences (e.g., ≥700) was applied for keyword co-occurrence analysis.

These thresholds were selected to focus on the most influential entities while maintaining network clarity.

Before analysis, the dataset was cleaned to improve accuracy. This included

- Merging author name variants (e.g., differences in initials or spelling).
- Normalising keywords by combining synonyms and singular/plural forms using a thesaurus file.

## 4. Publication Trends

**Figure 2:**
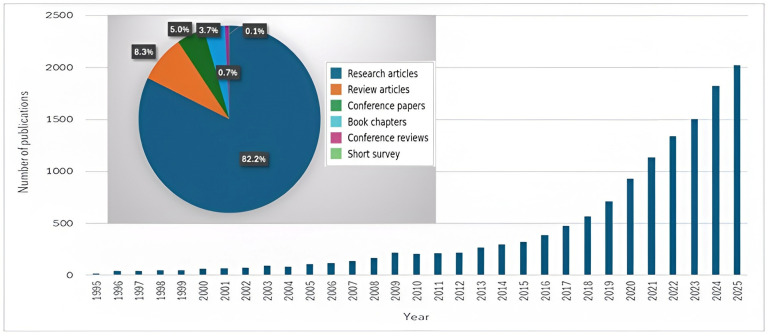
Number of published articles on “biopolymer-based materials in wastewater” based on the Scopus database (accessed on 20 November 2025).

The publication trend from 1995 to 2025 shows a clear and continuous rise in research output related to biopolymer-based materials for wastewater treatment, based on the Scopus query (chitosan OR alginate OR cellulose OR starch OR lignin OR pectin OR agar OR biopolymer wastewater treatment). Figure 2 illustrates both the annual growth in publications and the distribution of document types. While research articles dominate the dataset, review articles show a gradual increase, indicating a maturing research field with growing emphasis on synthesis and consolidation of knowledge. Early years (1995–2005) show very limited activity, with publications growing slowly from 17 documents in 1995 to just over 100 by 2005. This period reflects the foundational phase of biopolymer research, when the application of natural polymers in environmental remediation was still emerging.

A noticeable increase occurs from 2006 to around 2015, where annual publications steadily rise from 116 to 323 publications. This reflects growing scientific interest in sustainable materials, green chemistry, and low-cost biopolymers. The modification, characterization, and use of natural polymers in wastewater treatment studies became increasingly well-established during this time, and multidisciplinary research grew.

The most substantial growth appears from 2016, going up, marking the rapid expansion phase of the field. Publications climbed dramatically from 388 papers in 2016 to 1822 in 2024, with a peak production of 2023 documents in 2025. This growth may be associated with increasing global interest in sustainable water treatment solutions, although this relationship is not directly demonstrated by the bibliometric data. Overall, this trend indicates that biopolymer-based wastewater treatment has evolved into a significant and rapidly growing field of study.

## 5. Results

### 5.1. Publishing Sources with Citations

This bibliometric study discovered a total of 1384 source titles. A minimum threshold of 110 documents per source was applied; only 21 journals met the inclusion criteria. Among these, *Bioresource Technology* is the most prominent, with a contribution of 462 documents and 41,545 citations, followed by the *Chemical Engineering Journal* (258 documents; 20,026 citations), *Water Research* (272 documents; 32,385 citations), and the *Journal of Hazardous Materials* (241 documents; 30,221 citations). Other influential sources include *Chemosphere*, the *International Journal of Biological Macromolecules*, *Science of the Total Environment*, *Desalination*, the *Journal of Cleaner Production*, and *Separation and Purification Technology*, each accumulating more than 7000 citations. These journals serve as the primary publication venues for breakthroughs in biopolymer-based materials, environmental engineering, and wastewater treatment technology and may also suggest the interdisciplinary nature of the field. Table 2 lists the top 10 influential journals.

### 5.2. Most Contributing Countries

Out of 134 countries analysed, 51 met the minimum threshold of 50 documents, highlighting significant contributions to biopolymer-based wastewater treatment research (see the top 10 in Table 3). The analysis of publication and citation data reveals which countries are experts in biopolymer-based wastewater treatment research. China leads with 3809 papers and 159,611 citations, followed by India (1573 documents, 66,542 citations) and the United States (817 documents, 53,950 citations). The dominance of China, followed by India and the United States, may reflect a combination of factors, including increased national investment in environmental research, strong publication output in materials science, and the prioritisation of water treatment technologies in rapidly industrialising regions. Other noteworthy contributions include Malaysia, South Korea, Canada, Saudi Arabia, Egypt, Spain, and Australia. The large citation counts of these countries show that their research outputs have a considerable influence, with China dominating due to its emphasis on environmental issues and wastewater management. Overall, Asia and North America are the most productive and influential in this subject.

### 5.3. Top Organisations/Institutions

Applying a minimum criterion of 25 publications per organisation to the 13,720 documents in the bibliometric dataset yielded 21 institutions that matched the selection criteria. These organisations are the most prolific research centres in the sector, with China leading the way with many high-output universities and state-key laboratories, demonstrating the country’s strong and expanding investment in advanced environmental and materials research. Notably, the University of Chinese Academy of Sciences emerged as the leading university in terms of publications, followed by notable contributors such as Universiti Teknologi Malaysia, Guangxi University, and Jiangsu University (see Table 4). Although the representation of Malaysian and Netherlands institutions is limited, it indicates the worldwide distribution of expertise and collaborative effort in the topic. The concentration of leading institutions within specific regions may suggest the presence of established research specialising in biopolymer-based materials. This may be attributed to access to funding, infrastructure, and collaborative networks.

### 5.4. Co-Authorship Network

According to the co-authorship study, 25 authors out of a total of 40,671 authors met the requirement of at least 20 publications, showing that only a tiny elite group represents the field’s core collaborative structure. Zhang, Jian has the highest total link strength (TLS = 5), a metric used to assess the overall connectivity of an item within a scientific network, indicating that he is the most connected researcher in this collaboration network. The relatively low TLS values observed for several authors, including some highly cited contributors, suggest that the field may be characterised by a degree of fragmentation, with multiple research groups operating independently rather than within strongly interconnected collaboration networks. The presence of highly cited authors with TLS = 0 may indicate limited co-authorship within the defined network thresholds. See the summary in Table 5 below.

**Figure 3:**
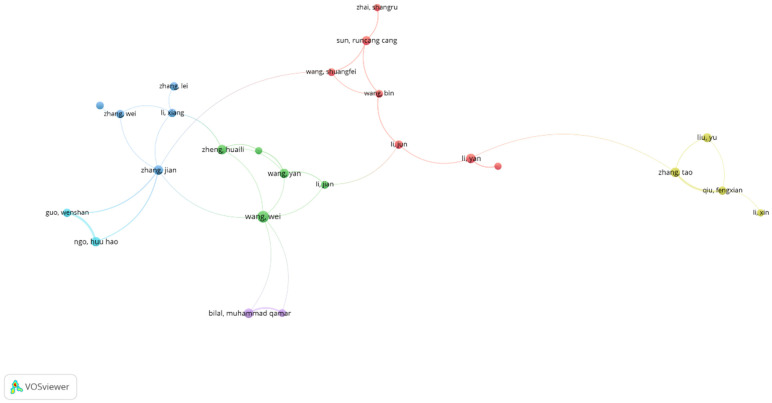
Visualisation of author collaboration patterns.

The co-authorship network depicts collaborative trends among authors in the area. Each node on the map symbolises an individual author, and its size indicates their publishing output. Authors who interact more frequently are positioned closer together (see Figure 3), and the thickness of the connecting lines shows the strength of their collaborations. Different colours reflect various cooperation clusters, indicating groups of writers that collaborate more frequently [69].

### 5.5. Most Cited Authors

Only 16 of the 40,671 authors found in the sample had at least 3000 citations. In this analysis, author influence is measured using total citations rather than publication count, because citations better represent research exposure, impact, and academic influence.

Among these high-impact authors, Crini, Gregório is the leading contributor with 11,304 citations, followed by Wan Ngah, Wan Saime Wan (4519 citations), and Yu, Hanqing (4458 citations) (see Table 6). Regardless of publication number, these authors represent the intellectual core of the subject, influencing the direction of research on biopolymer-based materials for wastewater treatment.

### 5.6. Most Cited Articles

It is important to evaluate the most cited and influential articles after the most cited authors in bibliometric studies. Herein, 14 of 11,680 papers have at least 1600 citations. The most prominent contributors were identified based on their citation impact rather than publication numbers. Crini (2006) is the most referenced paper, with 4130 citations. His article reviews the cost-effective adsorption strategies for removing non-biodegradable pollutants, particularly dyes, highlighting the growing use of low-cost natural and waste-derived sorbents as alternatives to activated carbon and emphasising chitosan as a highly promising material for environmental remediation [70]. The second contributing article was authored by Babel (2003), with 3171 citations. The article discusses the use of low-cost materials like chitosan, zeolites, lignin, and industrial wastes as alternatives to activated carbon for heavy metal removal, highlighting their exceptional adsorption capacities and that the chemical modification plays a major role in adsorption [71]. Gupta and Suhas (2009) had the third most influential articles, with 3151 citations. They reviewed low-cost alternative adsorbents (LCAs), including natural, industrial, and synthetic waste materials as efficient, economical options for dye removal, summarising their advantages, adsorption performance, and comparison with other treatment methods while outlining key insights and future research needs [72]. Other significant contributions are Bailey (1999) (2997 citations), who studies low-cost sorbents for metal remediation [73], and Sheng et al. (2010), with 2853 citations, who investigated extracellular polymeric substances (EPS) in biological treatment systems [74].

Notably, the most highly cited articles are mainly broad reviews on low-cost adsorbents, dye removal, and heavy metal adsorption rather than specifically on cross-biopolymer systems. This suggests that the field is more strongly rooted in general wastewater treatment and adsorption research than in a distinct, unified biopolymer-based materials domain. Table 7 summarises the most influential articles with their respective author(s).

### 5.7. Co-Occurrence Network

The co-occurrence of keywords was examined from all papers included in this study to uncover research hotspots, interrelated topics, and emerging trends in biopolymer-based wastewater treatment materials. From a total of 49,974 extracted keywords, those that appeared at least 700 times were selected, resulting in 43 high-frequency keywords for network analysis. Keyword mapping was performed using VOSviewer. Colour variations depict cluster groups, which represent theme intensities in the research subject. Circles positioned closer together represent stronger keyword relationships. The colour of each circle shows how keywords within the same cluster are interconnected [75].

**Figure 4:**
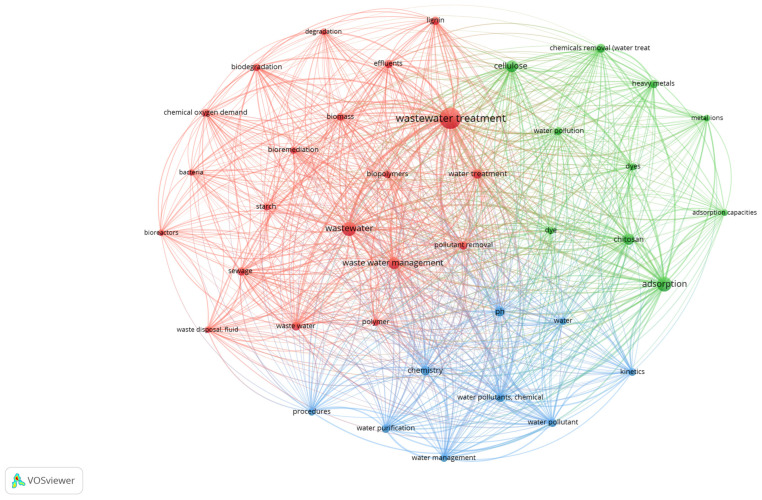
Co-occurrence network of high-frequency keywords (≥700 occurrences) related to biopolymer-based materials in wastewater treatment (1995–2025), generated using VOSviewer.

To investigate the progress, focus of study areas, and conceptual links in this domain, both author keywords and keywords-plus were normalised to unify plural/singular forms and combine synonymous terms using a thesaurus. As shown in Figure 4, “wastewater treatment” is the most common term, with 8684 occurrences and a total link strength of 47,475, suggesting its importance in this research environment. Other important phrases are “adsorption” (3586; TLS = 23,606), “wastewater” (3874; TLS = 29,128), “cellulose” (2317; TLS = 14,152), “chitosan” (2156; TLS = 12,234), and “water pollutants, chemical” (1272; TLS = 14,211) (see Table 8). These significant connection strengths show the ongoing emphasis on pollution removal, adsorption methods, and the usage of natural biopolymers such as cellulose, chitosan, lignin, and starch. Overall, the network demonstrates how biopolymer-based materials have become central to wastewater treatment.

## 6. Conclusions, Future Work, and Recommendations

This review article presents a complete bibliometric assessment of biopolymer-based materials in wastewater treatment, comprising 13,720 Scopus-indexed papers between 1995 and 2025. The study reveals a considerable global interest in sustainable wastewater technology. The key findings are summarised as follows:

- A significant increase in publication output was observed, particularly after 2016, indicating growing research interest in sustainable water treatment technologies.
- China, India, and the United States emerged as the most productive and influential contributors, reflecting strong research activity in environmental materials science.
- Leading journals are primarily positioned at the intersection of materials science and environmental engineering, highlighting the interdisciplinary nature of the field.
- Co-authorship analysis revealed relatively fragmented collaboration networks, suggesting the presence of multiple independent research groups and opportunities for stronger global collaboration.
- Citation analysis showed that highly cited works are largely dominated by broad review articles on adsorption and wastewater treatment, indicating that the field is intellectually anchored in the wider environmental remediation literature.
- Keyword co-occurrence analysis identified adsorption, wastewater treatment, chitosan, and cellulose as dominant research themes, with increasing attention given to composite materials and functional modifications.

From a broader perspective, these findings suggest that while biopolymer-based materials represent an important and growing area of research, the field is still evolving toward a more integrated and cohesive research structure.

Future research directions, informed by the broader literature context, may include

- Strengthening interdisciplinary and international collaboration networks;
- Advancing the integration of biopolymer materials into scalable and real-world applications.

## Figures and Tables

**Table 1:** Summary of biopolymer-based materials for wastewater treatment.

Biopolymer	Pollutants Removed	Mechanism	Adsorption Capacity (mg/g)	Reusability (Cycles)	References
Cellulose-based					
CBBAS (cellulose beads from bleached almond shell)	Cu (II)	Chelation	128.24	4	[26]
CCBG-g-PDMDAAC3.0 (DMDAAC-grafted chitosan/genipin/cellulose hydrogel beads)	Reactive Red 195	Electrostatic attraction	1333.52	5	[27]
CCBG-g-PDMDAAC3.0 (DMDAAC-grafted chitosan/genipin/cellulose hydrogel beads)	Methyl Orange	Electrostatic attraction	190.48	5	[27]
GT-cellulose (GTMAC-modified hemp cellulose)	Methyl Orange	Electrostatic attraction	76.9	-	[28]
Cationic cellulose (periodate oxidation + Girard’s reagent T)	Eriochrome Cyanine R (ECR)	Electrostatic attraction	65.33	5	[29]
Cationic cellulose fibre (GTMAC-modified cellulose pulp)	Reactive Black 5	Electrostatic attraction	1010	5	[30]
Alkali-scoured modified rice husk	Congo Red	Surface adsorption	93.8	-	[31]
Nascent rice husk	Crystal Violet	Electrostatic interaction	25.46	-	[32]
Carboxymethyl cellulose–alginic acid–polyethyleneimine	Cr(VI)	Chelation	293.26	5	[33]
PEI-modified cellulose	Methyl Blue	Electrostatic attraction and hydrogen bonding	1550.55	8	[34]
PEI-modified cellulose	Rose Bengal	Electrostatic attraction and hydrogen bonding	467.95	8	[34]
Chitosan-based					
Ammonium-modified chitosan composite	Congo Red	Electrostatic interactions and hydrogen bonding	1261.64	5	[35]
Chitosan-based composite microspheres	Eriochrome Black T dye	Electrostatic interactions	317.21	-	[36]
Nitrilotriacetic acid-modified magnetic chitosan microspheres	Tetracycline	π–π interactions, hydrogen bonding	625.52	5	[37]
Amine-thiourea-modified magnetic chitosan hydrogel	Ce (III)	Chelation, electrostatic interaction	156	5	[38]
Chitosan film	As(V)	Electrostatic interaction	15.23	4	[39]
Alginate-based					
Calcium alginate hydrogel beads	Methyl Violet	Electrostatic attraction	889	-	[40]
Alginate-coated perlite beads	Methylene Blue	Electrostatic attraction	104.10	5	[41]
ZnO nanoparticle-embedded Sodium alginate membrane	Methylene Blue	Electrostatic attraction and hydrogen bonding	746.00	3	[42]
Magnetic sodium alginate–zirconium(IV) beads	Pb (II)	Complexation	333.33	10	[43]
Pectin-based					
Low methoxy pectin–guar gum hybrid beads	Pb (II)	Ion exchange and complexation	104.8	-	[44]
Calcium pectate gel beads	Hg (II)	Ion exchange and complexation	340	-	[45]
Pectin microgel particles	Methylene Blue	Electrostatic attraction	284.09	3	[46]
Chitosan–pectin gel beads	Cu (II)	Complexation	169.4	5	[47]
Chitosan–pectin gel beads	Cd (II)	Complexation	177.6	5	[47]
Other biopolymer-based					
Crosslinked porous starch	Methylene Blue	Electrostatic attraction	9.46	-	[48]
Crosslinked starch	Pb (II)	Chelation	433	-	[49]
Crosslinked starch	Cu (II)	Chelation	135	-	[49]
Lignin-based polyurethane Foam	Methylene Green	Electrostatic attraction, π–π interactions, hydrogen bonding	80	20	[50]
Agar-APTES cryogels	Cr (III)	Ion exchange, chelation, electrostatic attraction	52.58	-	[51]

**Table 2:** Top-cited journals identified in the bibliometric analysis.

Rank	Source	Documents	Citations
1	*Bioresource Technology*	462	41,545
2	*Water Research*	272	32,385
3	*Journal of Hazardous Materials*	241	30,221
4	*Chemical Engineering Journal*	258	20,026
5	*Science of the Total Environment*	242	12,016
6	*International Journal of Biological Macromolecules*	382	14,401
7	*Chemosphere*	305	16,325
8	*Journal of Environmental Management*	188	17,560
9	*Carbohydrate Polymers*	174	17,871
10	*Separation and Purification Technology*	200	8710

**Table 3:** Top 10 most cited countries in bipolymer-based wastewater treatment research.

Rank	Country	Documents	Citations
1	China	3809	159,611
2	India	1573	66,542
3	United States	817	53,950
4	Saudi Arabia	394	15,787
5	Malaysia	499	23,741
6	South Korea	397	20,094
7	Canada	398	20,545
8	Egypt	412	14,094
9	Spain	349	18,354
10	Australia	296	15,430

**Table 4:** Leading 10 research institutions meeting the 25-document threshold.

Rank	Organisation/Institution	Country	Documents	Citations
1	University of Chinese Academy of Sciences	China	87	3351
2	Universiti Teknologi Malaysia (Johor Bahru)	Malaysia	75	2757
3	Guangxi University, Nanning	China	62	1934
4	Jiangsu University, Zhenjiang	China	61	1983
5	State Key Laboratory of Pollution Control Technology	China	43	1377
6	State Key Laboratory of Biobased Materials	China	42	1192
7	Department of Biotechnology, Delft University of Technology	Netherlands	40	2065
8	Harbin Institute of Technology (Main Campus)	China	34	1984
9	School of Environment, Harbin Institute of Technology	China	33	1339
10	Tongji University, Shanghai	China	32	2749

**Table 5:** Top 10 authors ranked by total link strength in the co-authorship network.

Rank	Author	Documents	Citations	Total Link Strength (TLS)
1	Zhang, Jian	25	1148	5
2	Ngo, Huu Hao	24	2476	4
3	Wang, Wei	35	1412	3
4	Li, Yan	25	793	1
5	Zhang, Tao	24	865	1
6	Zheng, Huaili	24	1353	1
7	Añón, J.C.R.	47	3814	0
8	Crini, Grégorio	24	11,304	0
9	Fatehi, Pedram	27	518	0
10	Sillanpää, Mika A.	27	3122	0

**Table 6:** Top 10 most cited authors.

Rank	Author	Documents	Citations
1	Crini, Gregório	24	11,304
2	Wan Ngah, Wan Saime Wan	6	4519
3	Yu, Hanqing	14	4458
4	Kurniawan, Tonni Agustiono	9	4374
5	Babel, Sandhya	2	4014
6	Gupta, Vinod Kumar A.	6	3958
7	Li, Xiaoyan	15	3951
8	Añón, J. C. R.	47	3814
9	Megat Hanafiah, Megat Ahmad Kamal	3	3637
10	Elimelech, Menachem	11	3633

**Table 7:** Summary of highly cited articles and their topics.

Rank	Article (First Author, Year)	Citations	Topic/Focus
1	Crini (2006)	4130	Non-conventional low-cost adsorbents for dye removal: A review
2	Babel (2003)	3171	Low-cost adsorbents for heavy metals uptake from contaminated water: A review
3	Gupta and Suhas (2009)	3151	Application of low-cost adsorbents for dye removal—A review
4	Bailey (1999)	2997	A review of potentially low-cost sorbents for heavy metals
5	Sheng (2010)	2853	Extracellular polymeric substances (EPS) of microbial aggregates in biological wastewater treatment systems: A review
6	Wan Ngah (2011)	2710	Adsorption of dyes and heavy metal ions by chitosan composites: A review
7	Haritash (2009)	2650	Biodegradation aspects of Polycyclic Aromatic Hydrocarbons (PAHs): A review
8	Crini (2008)	2152	Application of chitosan, a natural aminopolysaccharide, for dye removal from aqueous solutions by adsorption processes using batch studies: A review of recent literature

**Table 8:** Top 12 frequently used keywords in the research of biopolymer-based materials in wastewater treatment (1995–2025).

Rank	Keyword	Occurrences	Total Link Strength
1	wastewater treatment	8684	169,734
2	wastewater	3874	97,083
3	adsorption	3586	57,873
4	pH	1473	49,786
5	cellulose	2317	48,261
6	chemistry	1483	48,183
7	water pollutants, chemical	1272	43,271
8	water pollutant	1227	42,244
9	chitosan	2156	39,853
10	sewage	1219	36,001
11	pollutant removal	1177	33,801
12	dyes	1054	31,732

## Data Availability

No new data were created or analysed in this study. Data sharing is not applicable to this article.
